# Antidepressants for prevention of severe COVID-19, Long COVID and outlook for other viral diseases

**DOI:** 10.3389/fmed.2024.1305184

**Published:** 2024-02-20

**Authors:** Udo Bonnet, Georg Juckel, Jens Kuhn

**Affiliations:** ^1^Department of Mental Health, Evangelisches Krankenhaus Castrop-Rauxel, Academic Teaching Hospital of the University of Duisburg/Essen, Castrop-Rauxel, Germany; ^2^Department of Psychiatry and Psychotherapy, Faculty of Medicine, Landschaftsverband Rheinland-Hospital Essen, University of Duisburg-Essen, Essen, Germany; ^3^Department of Psychiatry, Psychotherapy and Preventive Medicine, LWL University Hospital, Ruhr University Bochum, Bochum, Germany; ^4^Department of Psychiatry and Psychotherapy, University Hospital Cologne, Cologne, Germany; ^5^Alexianer Hospital Cologne, Cologne, Germany

**Keywords:** antidepressants, resilience, prevention, COVID-19, Long COVID, Post COVID, PACS

## Introduction

Even though the global SARS-CoV-2 pandemic is considered to be over for the time being, there are fears of more frequent infections with the virus and new mutations in the coming winter months. In this context, one surprising lesson from the outgoing suffer seems to be related to antidepressants (AD), which might have the potential to be beneficial for prevention of SARS-CoV-2 infections and critical COVID-19 outcomes (1, 2). The vast majority of large-scale retrospective studies evaluating more 20000 patients point into this direction (Table 1). Especially selective serotonin reuptake inhibitors (SSRI) and serotonin–norepinephrine reuptake inhibitors (SNRI) seem to be efficient in this context. It is not clear whether this advantage is specific only for serotonergic AD as these are by far the most commonly prescribed AD or for the whole AD substance class itself, which comprised also AD with no direct serotonergic potential (e.g. bupropion—however being indirectly involved in the regulation of neuronal serotonin activity as found in rat brain) (14). The underlying biological mechanisms are assumed to be more sophisticated. In addition to direct inhibitory effects on several steps of the viral infection process itself (Supplementary Figure 1), the mitigation of the remarkable and sustainable pro-inflammatory cellular response to SARS-CoV-2 (“hyperinflammation”) is favored (1, 2). Within the latter, the most prominent mechanisms are activation of the intracellular sigma-1 receptor – IRE 1-System by SSRI (first demonstrated for fluvoxamine) (15) and the functional inhibition of acid sphingomyelinase (FIASMA) by most AD, including SSRI, SNRI, tricyclics, mirtazapine, trazodone and bupropion (1, 16).

**Table 1:** Brief overview of clinical studies testing the effectiveness/efficacy of antidepressants (AD) on COVID-19 severity/sequels (as of 09/25/2023 found in pubmed; https://pubmed.ncbi.nlm.nih.gov/).

**Meta-analyses**	**Placebo-controlled randomized studies**	**Prospective studies**	Retrospective large scale studies^*^
Vai et al. 2021: 0 AD; 10.1016/S2215-0366(21)00232-7	Lenze et al. 2020: + Fluvoxamine^**^; 10.1001/jama.2020.22760;	Seftel and Boulware 2021: + Fluvoxamine; 10.1093/ofid/ofab050	Oskotsky et al. 2021: + SSRI; 10.1001/jamanetworkopen.2021.33090
Firouzabadi et al. 2022: + SSRI/SNRI; 10.1002/hsr2.892	Lenze et al. 2021: (3) 0 Fluvoxamine^***^; http://clinicaltrials.gov/ct2/show/NCT04668950	Calusic et al. 2021:- + Fluvoxamine; 10.1111/bcp.15126	Fritz et al. 2022: (4) + AD (Dose Dependence); 10.1038/s41398-022-02109-3
Fico et al. 2022: + Fluvoxamine; 10.1016/j.euroneuro.2022.10.004	Reis et al. 2022: + Fluvoxamine; 10.1016/S2214-109X(21)00448-4	Pineda et al. 2022: (5) + Fluvoxamine; 10.3389/fphar.2022.1054644;	Stauning et al. 2023: - SSRI; 10.1016/j.cmi.2023.04.028
Nakhaee et al. 2022: + Fluvoxamine; 10.1371/journal.pone.0267423	Bramante et al. 2022 : (6) 0 Fluvoxamine (2x50 mg); 10.1056/NEJMoa2201662	Kirenga et al. 2023:15 + Fluvoxamine; 10.1038/s41380-023-02004-3	Visos-Varela et al. 2023: + SSRI; 10.1016/j.euroneuro.2023.03.011
Boretti 2022: (7) + Fluvoxamine; 10.1016/j.euroneuro.2022.12.001	Seo et al. 2022: (8) 0 Fluvoxamine (pneumonia)^****^; 10.3947/ic.2021.0142	Siripongboonsitti et al. 2023^***^: (9); 0 Fluvoxamine in combination with favinavir or flavinavir plus dexamethasone (standard immunomodulatory therapy); 10.1016/j.ijid.2023.06.018	Trkulja and Kodvanj 2023: + Fluvoxamine; 10.1007/s00228-023-03479-3
Lee et al. 2022: + Fluvoxamine; hospitalization rate; 10.1001/jamanetworkopen.2022.6269;	McCarthy et al. 2023: (10) 0 Fluvoxamine (2x50 mg); SARS-CoV-2 infection rate; 10.1001/jama.2022.24100	————————-	Schultebraucks et al. 2023: + AD; 10.1038/s41380-023-02049-4
Nyirenda et al. 2022: + Fluvoxamine; 10.1002/14651858.CD015391	Sedighi et al. 2023: (11) 0 Fluoxetine (pneumonia)^****^; 10.1002/npr2.12327		Ma et al. 2023: + Sertraline; 10.1016/j.euroneuro.2022.11.009
Bhuta et al. 2022: 0 Fluvoxamine; 10.1097/MJT.0000000000001496	————————–		Hasse et al. 2023: + SSRI, especially Sertraline; 10.1093/jphsr/rmad031
Lu et al. 2022: + Fluvoxamine; 10.1016/j.jiph.2022.10.010			Hoertel et al. 2023: (12) + AD; 10.3390/ph16081107
Wen et al. 2022: + Fluvoxamine; 10.1080/07853890.2022.2034936			————————–
Cobos-Campos et al. 2023: + SSRI/SNR; 10.1097/MJT.0000000000001618			
Vatvani et al. 2023: 0 Fluvoxamine; 10.1177/10600280231162243; see also the dose-response relationship discussion of Sánchez-Rico et al. 2023 in 10.1177/10600280231211304;			
Deng et al. 2023: + Fluvoxamine (dose dependence) and alternatively, fluoxetine; 10.1016/j.cmi.2023.01.010			
—————————			
**Meta-analyses**	**Placebo-controlled randomized studies**	**Prospective studies**	**Retrospective studies and case series**
**Long-COVID** ^*****^ **(mainly brain fog/fatigue, sensory overload, and anxiety/depression)**			
—————————	Bramante et al. 2022: 0 Fluvoxamine (2 × 50 mg); 10.1101/2022.12.21.22283753	Di Nicola et al. 2023: + Vortioxetine; 10.1016/j.euroneuro.2023.02.006	La Sala et al. 2023; + Tricyclic AD (2 Cases); 10.1097/JCP.0000000000001725
	Farahani et al. 2023: (+) Fluoxetine (Fatigue); 10.1186/s12879-023-08172-5	Fontera et al. 2022: + AD; 10.1371/journal.pone.0275274	Rus et al. 2023; + SSRI (*N* = 95); 10.1038/s41598-023-45072-9
	—————————	————————-	Sidky et al. 2023:; + SSRI (*N* = 17,933); preprint; 10.1101/2022.11.09.22282142
			—————————

^*^>20,000 pariticipants/e-health records (with the exception of Long COVID-studies). In pubmed, more than 10 further retrospective studies with < 20,000 participants supported the use of AD for reduction of SARS-CoV-2 infection rates as well as COVID-19 morbidity and mortality.

^**^The treatment trials with fluvoxamine tested usually daily doses of 2-3 x 100 mg over 10-14 days (Exceptions with lower doses: Bramate et al. (6): 10.1056/NEJMoa220166; McCarthy et al. (10); 10.1001/jama.2022.24100; Bramante et al. (2022): 10.1101/2022.12.21.22283753; here the daily lower doses were 2 x 50 mg).

^***^In both, the fluvoxamine and the control group, there were no deaths. This results in the assessment of “0” (see below) because the mortality rate was the primary endpoint of this study.

^****^No influence on COVID-19 pneumonia.

^*****^In accordance with the definition of the WHO we did not separate Long-COVID from Post-COVID. Long COVID (= Post COVID) is defined “as the continuation or development of new symptoms 3 months after the initial SARS-CoV-2 infection, with these symptoms lasting for at least 2 months with no other explanation” (13).

+ = Regarding COVID-19: Reduction of the morbidity (e.g., hospitalization rate, ventilation rate) and mortality by and with COVID-19 – regarding Long COVID: Reduction of the morbidity rate.

0 = No significant influence on the morbidity and mortality.

– = Increase in the mortality rate.

## Controlled clinical studies

For further evidence that AD might be beneficial for COVID-19 and also for its aftermath, the post-acute COVID-19 syndrome (PACS = Long COVID syndrome), prospective studies, placebo-controlled randomized controlled studies (RCT) and meta-analyses should be considered above all. As of 09/25/2023, literature research in pubmed revealed five prospective studies (plus 2 for Long COVID), 7 RCT (plus 2 for Long COVID) and 12 meta-analyses in this context (Table 1). Among the meta-analyses (incorporating primarily RCT and prospective studies, mostly with fluvoxamine), only three (one for any AD and two for fluvoxamine) showed no “anti-COVID-19 effect” (see these meta-analyses in bold letters in Table 1), but notably any negative effects on COVID-19 outcomes, which means that AD were not associated with a deterioration of COVID-19. A possible explanation for the lack of a “protective” effect could be following a dose-response relationship, i.e., that in the case of fluvoxamine, its tested dosage (2 x 50 mg/d) (6, 10) was simply too low. This assumption is supported by the results of a large retrospective study exploring the effects of AD on COVID-19 severity and mortality (*N* = 25 034) (4). Also bearing in mind the assumed mechanisms of action, which make the major effect in particular at the beginning of the infection plausible, the current evidence suggests that the earlier the AD were administered to patients exposed to SARS-CoV-2 or showing first mild-to-moderate COVID-19 symptoms, the more favorable was the outcome regarding SARS-CoV-2 infection rates, COVID-19 severity and related mortality (7, 17). On the other hand, AD seemed to have no relevant impact when they were added to the standard treatment of a full-blown COVID-19 pneumonia (Table 1) (8, 11). The tolerability of the AD (mostly fluvoxamine, sertraline and fluoxetine) usually added to the standard treatment (“add on”) of COVID-19, was reported to be generally well across all prospective, randomized controlled studies and meta-analyses presented in Table 1.

According to Long COVID, the clinical study situation is still limited, however, showing first preliminary promising results for AD (Table 1), even when these drugs were administered the first time during this condition (see retrospective studies and case series in Table 1) and not only specifically within the beginning of the SARS-CoV-2 infection.

## Discussion

In the light of GRADE (18), we think that the quality evidence for “add on” fluvoxamine for prevention of SARS-CoV-2 infections and severe COVID-19 has reached currently *the low-to-moderate level*. Thus, we support the utilization of this cheaply and easily available drug especially in regions where vaccination and approved “anti-COVID-19” immunomodulatory medications are far from being available. At this juncture, there are first promising results from prospective real-world studies carried out at Honduras (5) and Uganda (19). The same could be true for the whole substance class of AD according to the results of two large scale retrospective studies currently having tested the broadest spectrum of AD (4, 12).

As the pandemic is going to reach its end, it will be rather difficult to provide the next step necessary for further increasing the evidence quality, i.e., performing a prospective randomized controlled trial that compares the efficacy and tolerability of fluvoxamine or another AD with an approved drug for prevention of severe COVID-19. A most recent randomized prospective study found no superiority of fluvoxamine added to immunomodulatory standards (*N* = 134) (9). However, it should be outlined that in similar to the largest (although still unpublished) placebo-controlled clinical trial (*N* = 670) (3), there was a very low rate of hospitalization or intensive care and zero mortality in all groups which did not allow any sufficient differentiation according to severe COVID-19 conditions between the study groups (9).

With new mutations constantly emerging and vaccine development lagging behind we think, that the current evidence is sufficient enough to conduct a prospective study comparing an AD alone with a standard immunomodulatory therapy in mild and moderate COVID-19. The very promising, likely positive “anti-COVID-19” effect of AD, perhaps also against or for prevention of Long COVID (first results presented in Table 1) should stimulate further research on this drug-class for the treatment of other infectious diseases threatening the public health. Whether the underlying mechanism of action against Long COVID can also be suspected at an immunological level or is more a “conventional” primary AD effect [e.g., against Long COVID depression (20) or Long COVID fatigue (21)] still remains to be seen. To conclude and clarify, while we think to you have presented enough evidence to show that consideration can be given to trialing AD vs. therapy in mild-moderate COVID-19, there is currently not enough clinical evidence for extrapolating this to other infectious diseases.

## Outlook

There is growing evidence of a close and likely causal relationship between systemic inflammation (e.g., resulting from respiratory infections) and stress-related disorders, especially depressive states (22–25) (supporting the evolutionary concept of a “sickness behavior”) (26, 27). On the other hand, at least in our clinical experience, the susceptibility for infections of people in severe depressive states improved along with the recovery from depression. We are surprised to find no controlled clinical studies on this topic. In the light of the “anti-COVID” experiences with AD it is likely that anti-viral and anti-inflammatory properties of AD itself are crucially involved also in the recovery of those concomitant infections, beyond recovery from depression. In other words, beyond depression treatment, the “hidden” role of favorable immunomodulatory properties of AD in the treatment of moderate to severe depression should be underscored in the light of the above mentioned experiences during the pandemic. AD might be very helpful agents, not only in the treatment of cancer, but also for prevention and, perhaps also in the treatment of post-infection sequels. Besides antiviral properties, the strengthening of the cellular or tissue “resilience” against oxidative stress may play a key role (Supplementary Figure 1) (2). Anti-inflammatory AD properties include autophagy as a conserved strategy governing cellular energy and protein homeostasis, which might support beneficial effects of AD not only in severe depression itself, but also in other “stress-related” (often co-morbid) diseases, e.g., sleeping, anxiety and somatoform disorders, as well as anecdotally, in cancer and neurodegenerative diseases (27, 28). Their role as efflux pump inhibitors counterbalancing the cellular extrusion of antibacterial, anticonvulsant, psychiatric and anticancer medications could pose an additional advantage to limit the development of therapy resistance during the treatment with these drugs (29). Beyond COVID and depression, it is absolutely worth to conduct future repurposing studies on AD at least for further infectious diseases (27, 29, 30). Furthermore, investigating the extent to which a genetic predisposition influences the severity of COVID-19/Long-COVID (31) or other virus infection diseases remains to be challenging. In this regard, further insights on the role of AD in the development and course of those infection diseases could be expected.

## Author contributions

UB: Conceptualization, Data curation, Formal analysis, Investigation, Methodology, Project administration, Resources, Software, Visualization, Writing – original draft. GJ: Validation, Writing – review & editing. JK: Validation, Writing – review & editing.

## Supplementary material

Supplementary Figure 1 Cellular “anti-COVID”-mechanisms of antidepressants (AD): proven inhibitory effects of AD on distinct mechanisms triggering cell dysfunction or cell death in the SARS-COV-297–infection-cell stress response cascade are illustrated in the red boxes, according to Hoertel et al. and Bonnet and Juckel (1, 2).
