# Supplementary figures and images for: Antidepressants for prevention of severe COVID-19, Long COVID and outlook for other viral diseases
Source: Front Med (Lausanne). 2024 Feb 20;11:1305184. doi: 10.3389/fmed.2024.1305184 (PMC10912328; doi:10.3389/fmed.2024.1305184)

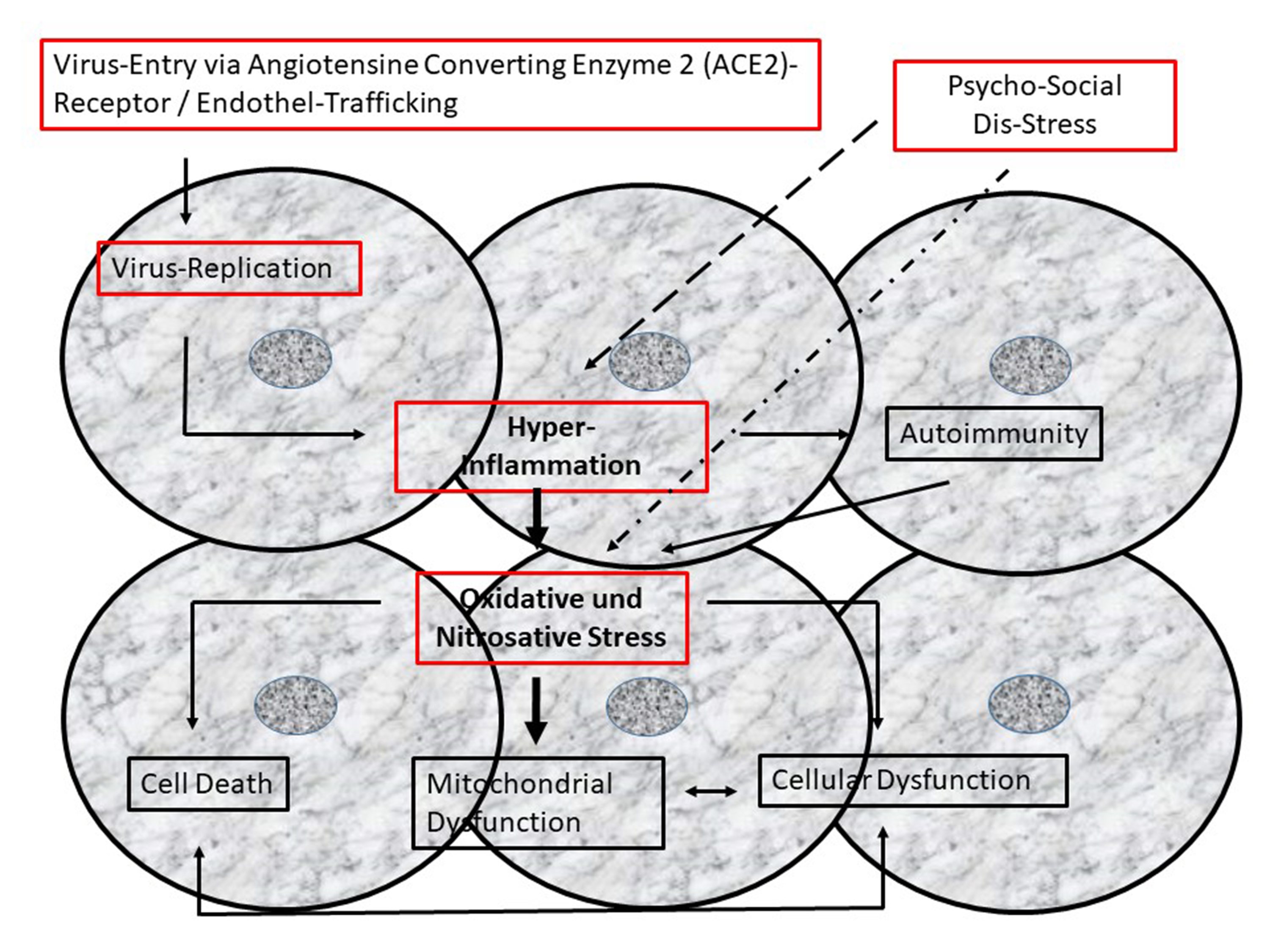

Supplement: Supplementary Figure 1 — Cellular “anti-COVID”-mechanisms of antidepressants (AD): proven inhibitory effects of AD on distinct mechanisms triggering cell dysfunction or cell death in the SARS-COV-297–infection-cell stress response cascade are illustrated in the red boxes, according to Hoertel et al. and Bonnet and Juckel (1, 2). [file Image_1.JPEG]
